# Colonization resistance: the role of gut microbiota in preventing *Salmonella* invasion and infection

**DOI:** 10.1080/19490976.2024.2424914

**Published:** 2024-11-08

**Authors:** Lei Deng, Shaohui Wang

**Affiliations:** a Shanghai Veterinary Research Institute, Chinese Academy of Agricultural Sciences, Shanghai, China; b Infectious Disease and Microbiome Program, Broad Institute of MIT and Harvard, Cambridge, MA, USA; c Center for Computational and Integrative Biology, Massachusetts General Hospital, Boston, MA, USA

**Keywords:** Gut microbiota, colonization resistance, *Salmonella*, immune response, dietary intervention

## Abstract

The human gastrointestinal tract is colonized by a complex microbial ecosystem, the gut microbiota, which is pivotal in maintaining host health and mediating resistance to diseases. This review delineates colonization resistance (CR), a critical defensive mechanism employed by the gut microbiota to safeguard against pathogenic bacterial invasions, notably by *Salmonella*. We detail the mechanisms through which the gut microbiota impedes *Salmonella* colonization, including nutrient competition, production of antimicrobial peptides, synthesis of microbial-derived metabolites, and modulation of the host immune response. Additionally, we examine how dietary interventions can influence these mechanisms, thereby augmenting the protective role of the gut microbiota. The review also discusses the sophisticated strategies utilized by *Salmonella* to overcome these microbial defenses. A thorough understanding of these complex interactions between microbial symbionts and pathogens is crucial for the development of innovative therapeutic strategies that enhance CR, aiming to prevent or treat microbial infections effectively.

## Introduction

The human gastrointestinal tract harbors a complex microbial ecosystem, known as the gut microbiota, which includes bacteria, viruses, fungi, and protozoa.^1,2^ This microbial community plays an indispensable role in host health, aiding in digestion, nutrient absorption, and immune function. It also protects against pathogenic invasions through a mechanism known as colonization resistance (CR).^3^ CR represents a multifaceted defense strategy where the native microbiota outcompetes pathogens for nutritional resources and ecological niches, produces antimicrobial agents, and modulates host immune responses.^4^ These interactions within the gut microbiota can effectively prevent colonization by enteric pathogens, such as *Salmonella*, offering a promising alternative to conventional antibiotic treatments.^5^

*Salmonella* spp., notorious for causing gastrointestinal and systemic infections, continue to pose a significant public health threat globally.^6^ These pathogens are responsible for two principal types of illnesses: gastroenteritis – typically foodborne and characterized by symptoms such as diarrhea, fever, and abdominal cramps – and typhoid fever, a more severe systemic infection caused by specific serotypes like *Salmonella* Typhi.^7,8^ In the United States, the Centers for Disease Control and Prevention (CDC) estimates that *Salmonella* is responsible for roughly 1.35 million infections and 26,500 hospitalizations each year, predominantly through contaminated food. Vulnerable populations, including the young, elderly, and immunocompromised, face higher risks of severe complications and increased mortality.^9^ Furthermore, *Salmonella* spp. also present considerable health threats to livestock, poultry, and occasionally pets, acting both as a primary pathogen and as a complicating factor in animals with preexisting illnesses.^10^

This review explores the dynamic interactions between the gut microbiota and *Salmonella* pathogens, with a particular focus on *Salmonella enterica* serovar (*S*.) Typhimurium, a leading cause of inflammatory diarrhea. We aim to provide a comprehensive overview of the various CR mechanisms that inhibit *Salmonella* colonization and infection and examine how *Salmonella* manipulates the gut microbiota to establish itself within the host. By detailing these complex interactions, this review seeks to highlight potential microbiota-targeted strategies that could bolster resistance against *Salmonella* and other enteric pathogens, thus paving the way for innovative infection control approaches.

### Direct mechanisms of CR in the gut microbiota

The gut microbiota employs distinct mechanisms to modulate its composition and resist pathogenic invasion, primarily through nutrient competition and interference competition. Nutrient competition is the battle for scarce resources such as carbohydrates, minerals, and electron acceptors within the gut. This competitive scenario is crucial for maintaining microbial balance by limiting the resources available to potential pathogens. Interference competition involves the production of bioactive compounds by certain microbes, which directly inhibit the growth or survival of competing species. These compounds include antimicrobial peptides and other metabolites that disrupt pathogen colonization. Furthermore, niche competition plays a pivotal role, where commensal bacteria colonize specific ecological niches, effectively blocking pathogens from establishing themselves by monopolizing critical spatial and resource-based niches.

#### Nutrient competition and CR

**Figure 1.**
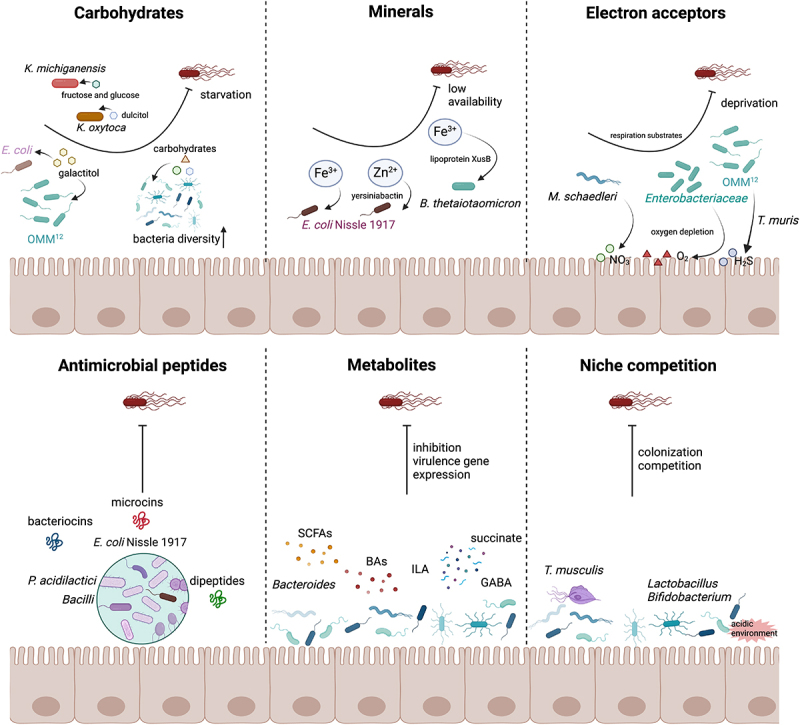
Direct mechanisms of CR in the gut microbiota. Multifaceted strategies employed by commensal bacteria to inhibit *Salmonella* colonization within the gut environment. Key mechanisms include competition for essential resources such as carbohydrates, minerals, and electron acceptors, effectively reducing their availability to pathogens. Commensal strains such as *E. coli* and members of the *Enterobacteriaceae* family produce antimicrobial peptides and other bioactive metabolites, which further hinder pathogen establishment. Additionally, these commensals occupy critical ecological niches, preventing pathogen access and colonization through spatial exclusion and metabolic dominance. The large red bacterium with flagella represents *Salmonella.*

Competition for nutrients constitutes a primary mechanism through which the gut microbiota confers against pathogens (Figure 1). Commensal bacteria, having adapted to efficiently utilize available nutrients, can outcompete pathogenic bacteria by depleting these resources to inhibit their establishment and proliferation.^11^ For instance, certain *Bacteroides* strains are adept at breaking down complex polysaccharides, which not only support their own growth but also enhance the stability of the entire gut microbiota ecosystem.^12^ Galactitol, a sugary alcohol derived from galactose, plays a pivotal role in microbial competition by serving as a crucial carbon source for specific bacteria.^13^ The competition for galactitol is pivotal, as the commensal *Escherichia coli* (*E. coli*) strain Mt1B1 can inhibit *S*. Typhimurium colonization by depleting this crucial substrate. Importantly, this effect is dependent on the microbial context, particularly the presence of the OMM^12^ microbial community, which significantly influences these competitive interactions.^14^ Additionally, during co-colonization with a different strain, *Salmonella* can utilize alternative carbon sources like galactitol or arabinose.^15^ Spragge et al. demonstrated that a diverse microbiota, consuming essential nutrients such as carbon sources needed by *S*. Typhimurium, enhances CR and may confer health benefits to the host.^16^ Moreover, *in vitro* co-cultures of *Klebsiella michiganensis* (*K. michiganensis*) and *S*. Typhimurium in minimal media supplemented with fructose or glucose showed that *K. michiganensis* not only inhibits the growth of *S*. Typhimurium but also hampers its expansion in the mouse gut, thereby prolonging host survival following *Salmonella* infection.^17^ Similarly, *Klebsiella oxytoca* (*K. oxytoca*) confers CR against *S*. Typhimurium by utilizing dulcitol, another sugar alcohol, in gnotobiotic mice.^18^

Minerals, such as iron and zinc, are critical for the growth and survival of both commensal and pathogenic bacteria. Gut microbiota have developed sophisticated strategies to sequester these essential minerals, thereby limiting their availability to pathogens.^19^ *E. coli* Nissle 1917 (EcN), for example, employs siderophores to tightly bind iron, thus reducing its availability to competing pathogens like *Salmonella*.^20^ In addition to iron acquisition, EcN also utilizes the siderophore yersiniabactin as a zincophore to enhance its colonization capacity in zinc-limited environments.^21^ Gut commensal *Bacteroides thetaiotaomicron* (*B. thetaiotaomicron*) acquires iron via siderophore-binding lipoprotein XusB and sustains its resilience in the inflamed gut by utilizing siderophores produced by other bacteria, including *Salmonella*.^22^

*S*. Typhimurium, a facultative anaerobic enteric pathogen, thrives in environments enriched with oxygen (O_2_) or alternative electron acceptors such as hydrogen sulfide (H₂S) and nitrate (NO_3_^−^), facilitating its anaerobic respiration.^23^ Endogenous *Enterobacteriaceae* can leverage aerobic metabolism to outcompete *Salmonella* by efficiently utilizing these electron acceptors.^24^ Specific consortia of gut microbiota, such as OMM^12^, capable of oxygen respiration, have demonstrated enhanced resistance to *S*. Typhimurium, achieving levels of resistance comparable to those provided by a complete microbiota.^25^ Moreover, the recently identified bacterium *Taurinivorans muris* (*T. muris*) utilizes taurine released from taurine-conjugated bile acids by the OMM^12^ community to produce H₂S, thereby bolstering CR against *S*. Typhimurium.^26^ Additionally, *Salmonella enterica* serovar Enteritidis exploits increased epithelial oxygenation to colonize neonatal chicks, supporting its growth through aerobic respiration. Conversely, a combination of *Enterobacteriaceae* and spore-forming bacteria can confer resistance to *S*. Enteritidis by competing for oxygen, a critical resource for bacterial aerobic processes.^27^ Microbiota-induced peroxisome proliferator – activated receptor γ (PPAR-γ) signaling is crucial for driving colonocyte β-oxidation, which lowers luminal oxygen levels, thereby limiting respiratory electron acceptors available to pathogenic *Escherichia* and *Salmonella*.^28^ *Mucispirillum schaedleri* (*M. schaedleri*), a nitrate-respiring commensal bacterium, inhibits *S*. Typhimurium by competing for NO_3_^−^ and interfering with its invasion gene expression.^29^ Although the competitive nutrient utilization by commensal bacteria to prevent *Salmonella* colonization has been extensively studied, translating these findings to clinical applications necessitates further research. This necessity arises not only from significant differences between human gut microbiota and those in experimental models but also from the more complex interactions among the host, microbiota, and pathogens.

#### Roles of microbial-derived antimicrobial peptides in CR

Antimicrobial peptides, including bacteriocins and microcins, are potent molecules produced by some commensal bacteria that play a critical role in inhibiting pathogen proliferation (Figure 1). For instance, bacteriocins produced by *Pediococcus acidilactici* (*P. acidilactici*) not only inhibit the biofilm formation of *S*. Typhimurium but also impede the growth of its planktonic cells.^30^ In comparison, probiotic *Bacilli* produce bacteriocins such as subtilosin A and subtilin, which specifically inhibit *Salmonella* biofilm formation without affecting the planktonic cells.^31^ Furthermore, microcins produced by the probiotic EcN target microbes expressing specific siderophore receptors during intestinal inflammation to inhibit the growth of *S*. Typhimurium without severely impacting the broader microbiota.^32^ Similarly, commensal *E. coli* limits *Salmonella* invasion during inflammation by employing a TonB-dependent uptake system to deliver microcins fused with siderophores.^33^ Additional examples include certain commensal microbes that produce dipeptides capable of inhibiting *Salmonella* growth.^34^ Therefore, antimicrobial peptides represent a powerful potential tool to enhance CR by selectively targeting pathogenic bacteria while preserving the beneficial commensal microbes.

#### Role of microbial metabolites in enhancing CR

Short-chain fatty acids (SCFAs) such as acetate, propionate, and butyrate are vital metabolites produced by the fermentation of dietary fibers by gut bacteria (Figure 1). These SCFAs are crucial for maintaining gut health and providing resistance against pathogens.^35,36^ It has been shown that acetic and butyric acids, isolated from fecal material, inhibit the growth of *Salmonella enteritidis* in vitro, particularly under anaerobic conditions and at pH levels typical of the mouse colon.^37^ Additionally, butyrate has been found to suppress *Salmonella* growth by altering the expression of genes related to pathogen virulence.^38^ Propionate plays an essential role in maintaining a balanced gut microbiota and conferring health benefits. It has been observed to inhibit *S*. Typhimurium LT2 by increasing sensitivity through elevated citrate synthase activity, leading to the production of toxic 2-methylcitrate under blocked conditions of the 2-methylcitric acid cycle.^39^ *Bacteroides* species, which produce propionate, provide CR against *S*. Typhimurium by disrupting intracellular pH homeostasis. This resistance can be enhanced through propionate administration or by transferring *Bacteroides*.^40^ Bile acids, synthesized from cholesterol in the liver and metabolized into secondary bile acids by the gut microbiota, possess antimicrobial properties.^41^ These acids inhibit *S*. Typhimurium by repressing the invasive pathogenicity island-1 (SPI-1) gene transcription and protein secretion, effectively initiating invasion only when bile concentrations decrease near epithelial cells.^42^ Both primary bile acid cholate and secondary bile acid deoxycholate inhibit the virulence factor SPI-1 expression in *S*. Typhimurium, mediated by the transcriptional activator HilD.^43^ Similarly, chenodeoxycholic acid effectively inhibits virulence gene expression of *S*. Typhimurium and epithelial cell invasion by directly binding to and inhibiting HilD.^44^ Tryptophan, an essential amino acid, is metabolized by gut bacteria into bioactive compounds such as indole derivatives, enhancing barrier functions and immune responses, thus providing resistance against pathogens.^45^ One of these microbial tryptophan metabolites, indole-3-lactic acid (ILA), can activate the AHR/IL-22 pathway, offering protection against *S*. Typhimurium infection.^46^ In addition, succinate, produced by gut microbiota, such as *Bacteroides*, *Prevotella*, and *Ruminococcus* species, plays a pivotal role in regulating host homeostasis and modulating inflammatory responses.^47^ Administration of succinate enhances the colonization of beneficial *Clostridia*, reduces intestinal loads of *S*. Typhimurium, and decreases oxygen concentration in the intestines.^48^ However, *S*. Typhimurium can exploit host-derived succinate to boost its antimicrobial resistance and virulence gene expression via the succinate uptake transporter DcuB.^49^ Gamma-aminobutyric acid (GABA), primarily known as a neurotransmitter in the central nervous system, along with enriched beneficial gut microbiota such as *Alistipes* and *Lactobacillus*, has demonstrated the ability to inhibit *Salmonella* infection in mouse models.^50^ The therapeutic potential of these microbial-derived metabolites in treating gut infections and enhancing CR represents a promising avenue for future research. Further exploration is needed to identify and characterize unknown metabolites produced by the gut microbiome, understand their interactions with host cells and pathogens, and evaluate their potential therapeutic applications.

#### Niche competition and microbial dominance

Niche competition refers to the process by which different species or organisms compete for the same limited resources within a specific ecological niche, thereby influencing the distribution and abundance of each species in the environment (Figure 1). While direct studies on niche competition between gut microbiota and *Salmonella* are limited, research has explored competition involving other members of the *Enterobacteriaceae* family, such as *Escherichia* and *Klebsiella* species. For instance, a consortium of 18 commensal strains, isolated from healthy individuals, effectively regulates gluconate availability to control ecological niches. This regulation promotes the decolonization of *Klebsiella* and *Escherichia* species, thereby reducing intestinal inflammation in mice.^51^ Increased complexity of the gut microbiota, characterized by a diverse and abundant microbial population, correlates with decreased susceptibility to colonization by pathogens such as *Salmonella enterica*. This resistance is partly attributed to the competitive exclusion provided by the established microbiota.^52^ *Lactobacillus* and *Bifidobacterium* are well known for their beneficial effects on gut health. These microbes lower the pH of the gut environment, creating conditions that are less hospitable to pathogens like *Salmonella*.^53,54^ Non-bacterial members of the gut microbiota also play significant roles in niche competition. For example, colonization by *Tritrichomonas musculis* (*T. musculis*) prior to infection with *S*. Typhimurium offers protective effects against intestinal inflammation and maintains epithelial cell integrity through inflammasome-dependent mechanisms.^55^ Furthermore, *T. musculis* colonization has been shown to enhance the efficacy of an oral vaccine against *S*. Typhimurium infection by promoting mucosal IgA responses in a Blt1-dependent manner.^56^ However, the mechanisms underlying niche competition are complex and involve a myriad of factors including metabolic interactions, immune modulation, and ecological dynamics within the gut microbiota. Comprehensive understanding of these interactions is essential for developing targeted strategies to enhance CR against pathogens. Further research is needed to elucidate these mechanisms and to leverage this knowledge for therapeutic interventions.

### Indirect mechanisms of CR in the gut microbiota

**Figure 2.**
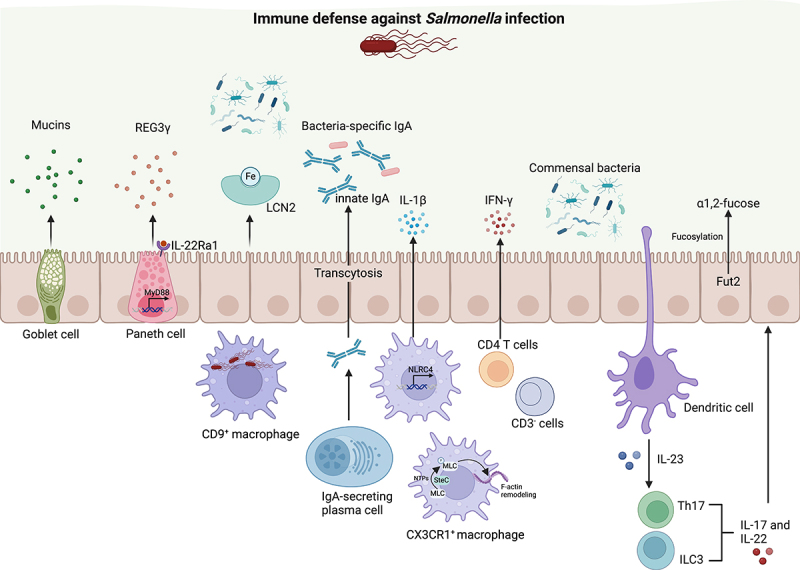
Indirect mechanisms of CR in the gut microbiota. Complex immune defenses in the gut counteract *Salmonella* infection by leveraging cellular and molecular components of the mucosal barrier. Goblet and Paneth cells secrete mucins and antimicrobial peptides such as REG3γ, critical for mucosal integrity. Bacteria-specific IgA, iron-sequestering proteins like LCN2, and cytokines including IL-1β, ifn-γ, IL-17, and IL-22 enhance immune responses. Commensal bacteria influence these defenses, enhancing mucosal barrier integrity and modulating immune cell signaling through pathways such as fucosylation mediated by Fut2. The large red bacterium with flagella represents *Salmonella.*

Apart from the direct inhibition of pathogen colonization by symbionts and their products, there are several indirect defense mechanisms that restrict the invasion and expansion of enteric pathogens in the gut. These mechanisms include the mucus layer and both innate and adaptive immune responses, all of which are induced or maintained by the gut microbiota to prevent pathogen colonization (Figure 2).

#### Maintenance of gut barrier integrity

The mucosal barrier is a critical component of the immune system, providing a physical and chemical shield to protect the body from harmful pathogens while maintaining a balance with beneficial microbes, and its dysfunction can lead to increased susceptibility to infections.^57^ Mucus, a highly adaptable protective barrier, varies in structure and function along the intestinal tract. In the gut, mucus forms physical barriers against pathogens like *S*. Typhimurium. However, regional variations exist – such as exposed epithelium in the cecum – which provide easier access for pathogens, making the cecum a primary site for *S*. Typhimurium infection.^58^ Muc2 mucin is a critical component of the mucosal barrier, primarily responsible for forming the protective mucus layer in the gut. An early study showed that Muc2-deficient mice are more susceptible to *S*. Typhimurium infection, exhibiting higher pathogen loads in the cecum and liver compared to their wild-type counterparts.^59^ The interaction between gut microbes and the mucus layer is vital for the development of this protective barrier. For instance, germ-free (GF) mice show markedly lower levels of Muc2 in their mucus compared to conventionally raised counterparts.^60^ Additionally, goblet cells within the epithelial lining secrete glycoproteins, or mucins, which form a robust mucus layer that acts as a shield against enteric pathogen infections. Paneth cells, a specialized epithelial lineage found in the small intestine, play a crucial role in maintaining intestinal homeostasis and defending the host by secreting antimicrobial molecules and reinforcing the gut barrier. Commensal bacteria stimulate the production of the antimicrobial lectin regenerating islet-derived protein 3γ (REG3γ) through the activation of MyD88 signaling in Paneth cells and other epithelial cells. This antimicrobial response helps limit the penetration of *S*. Typhimurium into host tissues, contributing to the prevention of infection and supporting gut health.^61^ *Salmonella* has developed mechanisms to breach this barrier, producing glycosyl hydrolases (GHs) that degrade terminal monosaccharides such as N-acetylneuraminic acid (Neu5Ac), galactose, mannose, and fucose. This enzymatic activity facilitates the pathogen’s attachment to the epithelial cell surface, aiding in its colonization and subsequent invasion.^62^ Preserving the integrity of the gut barrier and thwarting *Salmonella*‘s access to epithelial membranes presents a promising strategy for controlling infections not only in the gut but also across other epithelial surfaces.

#### Immune responses and microbiota interaction

The gut microbiota can modulate the host’s immune system to enhance its ability to clear pathogens by modulating the production of cytokines and the activation of immune cells.^35^ Lipocalin 2 (LCN2), a crucial antimicrobial protein, inhibits pathogen iron acquisition by sequestering bacterial iron-scavenging siderophores like enterobactin, with gut microbiota essential for inducing LCN2 expression in response to inflammation.^63^ Secretory Immunoglobulin A (sIgA) plays a crucial role in mucosal immunity by binding to and neutralizing pathogens, preventing their attachment and invasion of epithelial cells. IgA-mediated enchained growth is an important mechanism of restricting *S*. Typhimurium growth.^64^ Endt et al. reported that both gut microbiota and SIgA exert complementary functions in fighting *S*. Typhimurium infection, the microbiota provides CR and helps clear pathogens during initial infections, whereas sIgA offers protection from disease upon subsequent encounters with the same pathogen.^65^ Polyreactive IgAs enable the entry of noninvasive *S*. Typhimurium into Peyer’s patches independently of CX3CR1^+^ phagocytes, initiating a bacteria-specific IgA response that creates a positive feedback loop for further IgA production.^66^ Innate IgA also prevents epithelial cell invasion by *S*. Typhimurium and limits the spread of microbial pathogens within populations.^67,68^

IL-1β is a pro-inflammatory cytokine produced by activated macrophages and plays a key role in the inflammatory response. The presence of NLRC4 in macrophages contributes to the production of IL-1β, which helps distinguish pathogenic bacteria from commensal bacteria and enhances the clearance of *Salmonella* in mice.^69^ *Salmonella* also utilizes the effector protein SteC to promote CX3CR1^+^ macrophage migration and invasion through actin-dependent mechanisms, acting as an atypical kinase that phosphorylates host myosin light chain (MLC) using a broad range of NTPs, which is essential for its dissemination within the host.^70^ Hoffman et al. demonstrated that CD9^+^ macrophages provide an intracellular replication niche and detoxify oxidized lipids to promote *S*. Typhimurium expansion, while the depletion of CD9^+^ macrophages reduces CFU and extends mice survival.^71^ Although the microbiota–macrophage interaction remains underexplored in these studies, it is well established that the microbiota influences various immune cells, including macrophages, contributing to infection outcomes. Future studies are needed to investigate how microbial communities modulate macrophage function and subsequently shape infection dynamics and outcomes.

Interferon-gamma (IFN-γ) is another crucial cytokine for the immune response against intracellular pathogens like *Salmonella*. Certain commensals, including *Prevotellaceae*, *Porphyromonadaceae*, *Bacteroidaceae*, *S-24-7*, *Alcaligenaceae*, *Lactobacillaceae*, and *Lachnospiraceae*, can stimulate the innate cells and CD4^+^T cells to produce IFN-γ to control *Salmonella* infections.^72^ T helper 17 (Th17) and Th22 cells can maintain barrier surface integrity by containing commensal bacteria and expressing antimicrobial peptides against pathogens. IL-17A produced by gut-associated lymphoid tissue (GALT) is crucial for early protection against *S*. Typhimurium intestinal infection,^73^ and IL-17 receptor-deficient mice exhibit increased systemic *S*. Typhimurium dissemination.^74^ Similarly, IL-22, produced by innate lymphoid 3 cells (ILC3s), is crucial for the expression and maintenance of Fut2 and subsequent epithelial fucosylation in a commensal-dependent manner, which protects the host from the invasion of *S*. Typhimurium into the intestine.^75^ Paneth cell-specific IL-22Ra1 signaling is also crucial for preventing commensal dysbiosis, indicated by increased colonization by segmented filamentous bacteria (SFB), and for providing immunity against *Salmonella*.^76^ However, IL-22 also enhances *S*. Typhimurium colonization in inflamed intestines by suppressing commensal *Enterobacteriacea*e susceptible to antimicrobial proteins like LCN2 and calprotectin.^77^ Overall, the gut microbiota enhances host immune defenses by modulating cytokine production and immune cell activation, key to restricting pathogens like *Salmonella*. These mechanisms include the induction of antimicrobial proteins and the production of secretory IgA, which together help maintain the integrity of the gut barrier and modulate the immune response to prevent and control infection.

### Diet-microbiome-host immunity interactions

Diet plays a significant role in modulating the gut microbiome, with growing evidence indicating that dietary styles can influence susceptibility to various common infections.^11^ For example, vitamin A, obtained through the diet, is metabolized by commensal bacteria *Clostridia*, which suppress the expression of retinol dehydrogenase 7 (Rdh7) in intestinal epithelial cells to modulate the concentration of retinoic acid (RA). Deletion of Rdh7 in IECs diminished RA signaling in immune cells and IL-22-induced antimicrobial response, enhancing resistance to colonization by *S*. Typhimurium.^78^ Furthermore, vitamin A deficiency has been shown to increase susceptibility to disseminated non-typhoidal *Salmonella* infections in mice, as it disrupts terminal neutrophil maturation.^79^ Vitamin B12 deficiency leads to a decreased abundance of SCFA-producing bacteria, which in turn facilitates the expansion of *S*. Typhimurium. However, vitamin B12 supplementation can restore these phenotypes.^80^

Short-term reiterative switching to a high-fat diet can cause a disruption of intestinal microbiota and a temporary state of mucosal and systemic immune depression, increasing susceptibility to *S*. Typhimurium infections and a reduction of CD4^+^ T cell metabolic fitness and IL-17 cytokine production due to impaired mTOR activity.^81^ Both dietary and microbial factors influence the gut levels of conjugated linoleic acids (CLAs), which modulate CD4^+^CD8αα^+^ intraepithelial lymphocytes (IELs) by hepatocyte nuclear factor 4γ (HNF4γ) through interleukin-18 signaling in the small intestine. Axis of CLA-HNF4γ modulated CD4^+^CD8αα^+^ IEL plays a crucial role in the inhibition of *S*. Typhimurium infection.^82^ Fasting also protects mice from *S*. Typhimurium infection by suppressing its SPI-1 virulence program, a process dependent on the gut microbiome.^83^ A shift to a diet without fiber and oleic acid gavage promotes *S*. Typhimurium blooms and enteropathy, potentially due to the gut microbiota perturbations caused by these dietary changes.^84^

Microbiome maturation plays a critical role in the development of the immune system in infants. Disruptions of the intestinal microbiome during weaning impair immune system development and increase susceptibility to *Salmonella* infection.^85^ A recent study showed *S*. Typhimurium superspreader mice have distinct gut metabolomes compared with non-superspreaders, and diet-derived L-arabinose provides *S*. Typhimurium a competitive advantage in the gut, which requires an alpha-N-arabinofuranosidase that liberates L-arabinose from dietary polysaccharides.^86^ Microbiota-targeted diets hold promise for managing infectious diseases by modulating the gut microbiome to support immune function and inhibit pathogen colonization. Despite their current limited use in clinical settings, these specialized diets have the potential to significantly influence the future of infectious disease management through personalized dietary interventions tailored to individual microbiota compositions.

### Salmonella exploitation of the gut microbiota for colonization

**Figure 3.**
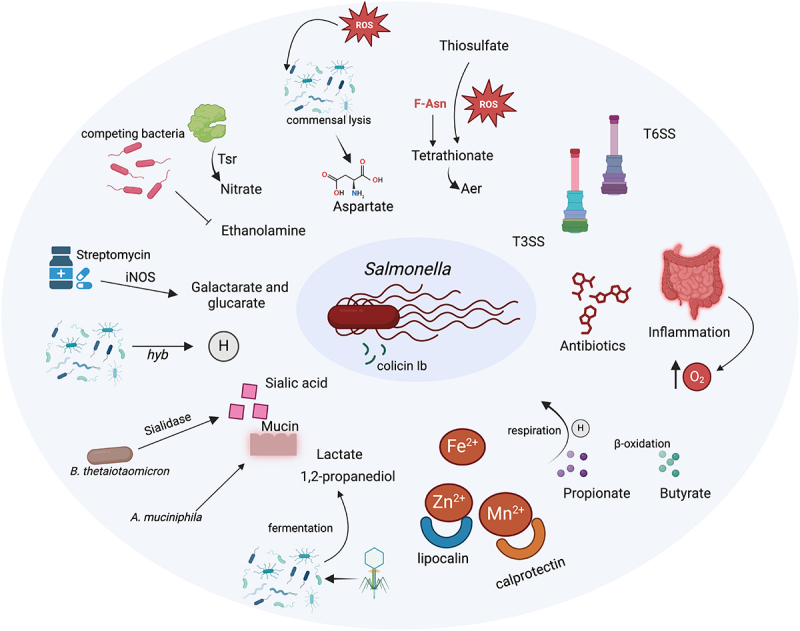
Mechanisms by which *S*. Typhimurium exploits host and microbiota to circumvent CR. Antibiotic treatments disrupt the microbiota, enhancing *S*. Typhimurium’s ability to thrive by altering the gut environment and reducing competition from commensal bacteria. In response to inflammation, the bacterium leverages altered gut conditions such as the conversion of thiosulfate to tetrathionate and increased oxygen levels, using these compounds as respiratory electron acceptors to outcompete anaerobic bacteria. T3SS and T6SS facilitates the injection of bacterial effectors into intestinal epithelial cells, disrupting the commensal balance and promoting pathogen expansion. *S*. Typhimurium also capitalizes on inflammation-induced metabolites like galactarate and glucarate and utilizes host-derived compounds such as ethanolamine and nitrate to support its growth. It metabolizes microbiota-derived substances including 1,2-propanediol, propionate, butyrate, and hydrogen. Additionally, the pathogen benefits from sialic acid released by mucin-degrading bacteria such as *B. thetaiotaomicron*. *S*. Typhimurium synthesizes siderophores and develops high-affinity transporters specifically for iron, zinc, and manganese, effectively circumventing host defense strategies designed to sequester these critical nutrients.

While the gut microbiota provides CR through the mechanisms discussed above, *Salmonella* can exploit these same interactions and resources to establish colonization within the gut (Figure 3). In a state of homeostasis, commensal microbes occupy various niches, preventing external bacteria from accessing essential nutrients and resources, thus serving as a primary barrier against pathogen colonization.^87^ The protective role of the commensal microbiota against invading pathogens was first recognized in the 1950s when Bohnhoff and colleagues demonstrated that a significantly smaller inoculum of *S*. Typhimurium could infect mice whose microbiota had been diminished by antibiotic treatment.^88^ Subsequent studies have consistently shown that perturbations in the microbiota caused by antibiotics create an environment conducive for *S*. Typhimurium to thrive in the gut.^89–92^ Moreover, intestinal inflammation can further facilitate the expansion of *S*. Typhimurium by suppressing the growth of commensal microbiota, demonstrating how pathogenic bacteria can exploit host and microbial dynamics to their advantage.^93^

The Type III secretion system (T3SS) and Type VI Secretion System (T6SS) are specialized protein export mechanisms used by gram-negative bacteria.^94^ T3SS functions by injecting effector proteins directly into host cells through a needlelike structure, facilitating bacterial invasion and immune evasion.^95^ SPI-1 T3SS initiates the *S*. Typhimurium infection by mediating epithelial invasion and triggering inflammation, while SPI-2 T3SS ensures intracellular survival, replication, and systemic spread.^96,97^ *S*. Typhimurium employs its T3SS to induce inflammation in the ileum and cecum of mice after surpassing the initial CR.^98^ T3SS–2 virulence, essential for the beneficial effects of Tsr-directed chemotaxis, is crucial for *S*. Typhimurium to exploit gut inflammation for colonization.^99^ In contrast, T6SS, which resembles a phage tail spike, is used by bacteria to pierce the membranes of neighboring cells and deliver enzymes or toxins, thereby either manipulating host cells or killing competing bacteria.^100,101^ In this context, the T6SS functions as an antibacterial weapon, where a bacterium injects multiple distinct toxins into a competitor cell. These toxins work together to degrade the competitor’s peptidoglycan cell wall in the periplasm or disrupt cellular functions in the cytoplasm, ultimately killing or inhibiting the target cell.^102^ *S*. Typhimurium utilizes the T6SS encoded within *Salmonella* pathogenicity island-6 (SPI-6), an essential virulence determinant for colonization, to kill commensal bacteria *K. oxytoca* to establish infection in the gut.^103^

*S*. Typhimurium exploits increased oxygen availability during gut inflammation, which suppresses native anaerobes and disrupts oxygen consumption by epithelial cells.^104^ Additionally, studies have demonstrated that *Salmonella* is able to utilize electron acceptors, inorganic nutrients, and sugars produced or metabolized by gut microbiota to promote its virulence.^105^ For instance, inflammation generates reactive oxygen species (ROS) that convert thiosulfate (S_2_O_3_^2−^) to tetrathionate (S_4_O_6_^2−^), a respiratory electron acceptor, which can be utilized by *S*. Typhimurium for respiration.^23^ Additional studies have shown that fructose-asparagine (F-Asn) is crucial for *S*. Typhimurium to metabolize S_4_O_6_^2−^ under anaerobic conditions in the inflamed intestine.^106^ Host-derived ROS also lysing commensal microbes increases aspartate availability, further promoting *S*. Typhimurium expansion.^107^ Methyl-accepting chemotaxis proteins (MCPs), such as Trg, Tsr, and Aer, act as respiratory electron acceptors, enhancing the fitness of *S*. Typhimurium in a mouse colitis mode.^108^ Moreover, *S*. Typhimurium can prompt the host to produce an additional respiratory electron acceptor NO_3_^−^, which serves as an energetically favorable electron acceptor, enhancing the growth of *S*. Typhimurium in the gut through anaerobic nitrate respiration.^109,110^ *S*. Typhimurium gains a growth advantage in the inflamed gut by respiring ethanolamine, which is provided by the host and not utilized by competing bacteria.^111^

Antibiotic treatment enhances the availability of oxidation products galactarate and glucarate by elevating the expression of the gene encoding inducible nitric oxide synthase (iNOS), which facilitated the expansion of *S*. Typhimurium.^112^ *S*. Typhimurium can exploit microbiota-derived hydrogen (H_2_) as an energy source, relying on Hyb hydrogenase.^113^ Certain commensal organisms, such as *B. thetaiotaomicron*, produce a mucin-degrading sialidase that releases sialic acid from mucins, which can be directly utilized by *S*. Typhimurium to support their own expansion in the gut.^114^ *Akkermansia muciniphila* (*A. muciniphila*), a mucin-degrading commensal bacterium, facilitates the expansion of *S*. Typhimurium by breaking down mucin and enhancing its access to the intestinal immune system.^115^ 1,2-Propanediol, also known as propylene glycol, is produced by the fermentation of dietary fibers by specific gut bacteria. *S*. Typhimurium utilizes both aerobic and anaerobic respiration to consume 1,2-propanediol to expand in a gut microbiota-dependent manner during intestinal inflammation.^116^ L-lactate, a key metabolite in cellular metabolism, serves as an energy source for various biological processes. *S*. Typhimurium can utilize host-derived L-lactate as a nutrient during gut infection.^117^

Bacteriophages are viruses that specifically infect and replicate within bacteria, playing a critical role in bacterial population dynamics and evolution. Using the simplified gnotobiotic mouse model (OMM^14^), Strempel et al. investigated the impact of two phage cocktails targeting *E. coli* and *Enterococcus faecalis* on their target strains and on CR. They found that the phages initially reduced the populations of their respective bacterial targets, which in turn increased susceptibility to *S*. Typhimurium infection. Notably, even 7 days after a single phage treatment – despite the bacterial populations returning to their original densities – OMM^14^ mice remained vulnerable to *S*. Typhimurium infection. Furthermore, phage treatment did not alter the overall microbiota composition or SCFA levels.^118^

While the gut microbiota employs a range of strategies to sequester essential minerals like iron, zinc, and manganese – thereby limiting their availability to pathogens—*S*. Typhimurium infection in rhesus macaques and mice triggers an enhanced production of the antimicrobial protein LCN2. This response, mediated by the *iroBCDE iroN* locus, provides *S*. Typhimurium with a competitive advantage, facilitating its proliferation in inflamed intestinal environments.^119^ Additionally, *S*. Typhimurium can overcome the zinc sequestration by calprotectin utilizing its high-affinity zinc transporter ZnuABC, enabling it to bypass this host defense mechanism.^120^ Moreover, *S*. Typhimurium deploys specialized metal transporters to acquire manganese, effectively evading calprotectin sequestration and outcompeting commensal bacteria in the inflamed gut.^121^ *S*. Typhimurium adapts its metabolism to exploit SCFAs in the inflamed gut environment. For instance, nitrate-dependent anaerobic respiration enables *S*. Typhimurium to metabolize propionate, supporting its intestinal expansion in the inflamed gut, a process dependent on the SPI-1 effector SopE and fueled by *Bacteroides*-derived propionate in gnotobiotic mice.^122^ Additionally, *S*. Typhimurium engages in anaerobic β-oxidation to harness microbiota-derived butyrate, further inducing intestinal inflammation.^123^ A recent study demonstrated that *S*. Typhimurium employs its virulence factors (T3SS) to overcome CR by utilizing aerobic respiration and mixed acid fermentation of simple sugars like glucose, leading to lower cecal levels of acetate and butyrate and increased epithelial oxygenation.^124^ *S*. Typhimurium adapts to the dynamic gut environment by strategically shifting between fermentation pathways, adjusting TCA cycle, and modulating energy production to ensure efficient colonization.^125^ *S*. Typhimurium produces colicin Ib (ColIb), a narrow-spectrum protein toxin that targets related *Enterobacteriaceae*, which conferred a competitive advantage to *S*. Typhimurium over sensitive *E. coli* strains in the inflamed gut.^126^ Elucidating the complex mechanisms through which *Salmonella* leverages the gut microbiota, host-secreted metabolites, and microbial-derived small molecules to circumvent CR is essential for developing targeted inhibition strategies. By comprehensively examining these intricate interactions, future research should aim to create microbiota-focused approaches that enhance resistance against *Salmonella* and other enteric pathogens.

## Summary and future prospects

The strategic modulation of CR by the gut microbiota presents a promising avenue to prevent infections and reduce dependence on antibiotics. This review has systematically explored the mechanisms – both direct and indirect – by which the gut microbiota combats pathogen invasion, with a specific focus on interactions with *Salmonella*. Direct mechanisms of CR include competition for essential nutrients and the synthesis of antimicrobial substances, which crucially inhibit the establishment of pathogens. Indirect mechanisms, more complex in nature, involve the modulation of the gut environment and immune responses. These mechanisms collectively foster a hostile environment for pathogenic bacteria while supporting the health of the host. Furthermore, dietary manipulation stands out as a viable strategy to influence microbiota composition and functionality, which can provide a noninvasive method to enhance natural defenses against pathogens.

However, CR within the gut microbiome is a multifaceted process involving niche competition, synthesis of metabolites and antimicrobial peptides, and modulation of host immune responses. This complex interaction between the microbiota, its metabolic products, and the immune system highlights the need for systematic studies of the gut microbiota-metabolite-immune axis to elucidate CR mechanisms and improve our understanding of gut health and pathogen defense. While CR has shown effectiveness in preclinical models against *Salmonella*, clinical translation requires consideration of complex factors such as host genetic variations and dietary habits that significantly influence the microbiota’s composition and function. Moreover, the gut hosts a diverse array of other microbiota components like viruses, fungi, and parasites, increasingly recognized for their roles in health modulation. Future research should also explore the metabolites from these organisms to understand their potential in regulating CR and the mechanisms involved.
